# A new skink of the genus *Scincella* Mittleman, 1950 (Squamata, Scincidae) from Dak Lak Province, Vietnam

**DOI:** 10.3897/zookeys.1275.178070

**Published:** 2026-03-31

**Authors:** Anh Van Pham, Dang Trong Do, Truong Quang Nguyen, Chung Van Hoang, Mai Hong Thi Nguyen, Minh Le, Minh Duc Le, Thomas Ziegler, Cuong The Pham

**Affiliations:** 1 Faculty of Environmental Sciences, University of Science, Vietnam National University, Hanoi, 334 Nguyen Trai Road, Hanoi 11416, Vietnam Institute of Zoology, University of Cologne Cologne Germany; 2 Faculty of Natural Sciences, Phu Yen University, Tuy Hoa Ward, Dak Lak Province, Vietnam Faculty of Environmental Sciences, University of Science, Vietnam National University Hanoi Vietnam; 3 Institute of Biology, Vietnam Academy of Science and Technology, 18 Hoang Quoc Viet Road, Hanoi 10072, Vietnam Central Institute for Natural Resources and Environmental Studies, Vietnam National University Hanoi Vietnam; 4 Graduate University of Science and Technology, Vietnam Academy of Science and Technology, 18 Hoang Quoc Viet Road, Hanoi 10072, Vietnam Graduate University of Science and Technology, Vietnam Academy of Science and Technology Hanoi Vietnam; 5 Central Institute for Natural Resources and Environmental Studies, Vietnam National University, Hanoi, 19 Le Thanh Tong, Hanoi 11021, Vietnam Institute of Biology, Vietnam Academy of Science and Technology Hanoi Vietnam; 6 Department of Herpetology, American Museum of Natural History, Central Park West at 79th Street, New York, New York 10024, USA Department of Herpetology, American Museum of Natural History New York United States of America; 7 Cologne Zoo, Riehler Straße 173, 50735, Cologne, Germany Faculty of Natural Sciences, Phu Yen University Tuy Hoa Ward Vietnam; 8 Institute of Zoology, University of Cologne, Zülpicher Straße 47b, 50674, Cologne, Germany Cologne Zoo Cologne Germany

**Keywords:** COI, Krong Trai Nature Reserve, molecular phylogeny, morphology, taxonomy

## Abstract

A new species of the genus *Scincella* Mittleman, 1950 is described from south-central Vietnam based on morphological and molecular evidence. *Scincella ngati***sp. nov**. is characterized by a combination of the following characters: size medium (SVL up to 48.3 mm); primary temporals two; external ear opening without lobules; loreals two; supralabials seven (rarely 8); infralabials six; enlarged nuchals, 0–2; midbody scales in 32–34 rows; dorsal scales smooth, in eight rows across the back; paravertebral scales 68–70, not widened; ventral scales in 64–68 rows; 10 or 11 smooth lamellae beneath finger IV and 16 or 17 beneath toe IV; toes not reaching the fingers when limbs adpressed along body; dorsal surface of body and tail bronze brown with a discontinuous black vertebral stripe, one scale wide, from middle of neck to tail base; a black stripe, two scales wide, interrupted by small pale spots, from nostril to eye and extending from posterior margin of eye along upper part of flank and tail base. In the phylogenetic analyses, the new species is recovered as an independent lineage with no clear sister taxon and shows at least 11.2% genetic divergence from other species in the genus based on a fragment of the mitochondrial COI gene.

## Introduction

The herpetofauna of Dak Lak Province has remained poorly studied. In recent years, seven new species have been described from Dak Lak Province, namely *Acanthosaura cuongi* Ngo, Le, Nguyen, Nguyen, Nguyen, Phan, Nguyen, Ziegler & Do, 2025 (Ngo et al. 2025); *A. grismeri* Le, Nguyen, Nguyen Ziegler, Do & Ngo, 2025 (Le et al. 2025); *Cyrtodactylus chumuensis* Ngo, Hormann, Le, Pham, Phung, Do, Ostrowski, Nguyen & Ziegler, 2023 (Ngo et al. 2023), *C. chuyangsinensis* Nguyen, Nguyen, Nguyen, Ha, Le, Grismer & Luu, 2025 (Nguyen et al. 2025b), *C. tayhoaensis* Do, Do, Ngo, Ziegler, Ngo, Nguyen & Pham, 2023 (Do et al. 2023), *Gekko phuyenensis* Nguyen, Nguyen, Orlov, Murphy & Nguyen, 2021 (Nguyen et al. 2021), *Gonyosoma iadinum* Poyarkov, Bragin, Idiiatullina, Tran, Le, David & Nguyen, 2025 (Poyarkov et al. 2025).

The genus *Scincella* Mittleman, 1950 currently comprises 51 recognized species with a wide distribution in Asia and America (Uetz et al. 2025). In Vietnam, Nguyen et al. (2009) listed three species of *Scincella*, viz. *S. doriae* (Boulenger), *S. melanosticta* (Boulenger), and *S. reevesii* (Gray). Since then, a total of 21 species of the genus have been documented from the country (Bragin et al. 2025b). During the last five years, eight new species have been discovered, namely *Scincella alia* Bragin, Zenin, Nguyen & Poyarkov, 2025 from Tuyen Quang Province (Bragin et al. 2025a); *S. auranticaudata* Nguyen, Nguyen, Le, Nguyen, Phan, Vo, Murphy & Che, 2025 from Lam Dong Province (Nguyen et al. 2025a); *S. balluca* Bragin, Zenin, Le, Nguyen, Nguyen, Bobrov & Poyarkov, 2025 from Dak Lak Province (Bragin et al. 2025b), *S. baraensis* Nguyen, Nguyen, Nguyen & Murphy from Binh Phuoc Province (Nguyen et al. 2020); *S. fansipanensis* Okabe, Motokawa, Koizumi, Nguyen, Nguyen & Bui, 2024 from Lao Cai Province (Okabe et al. 2024); *S. honbaensis* Nguyen, Nguyen, Le, Nguyen, Phan, Vo, Murphy & Che, 2025 from Lam Dong Province (Nguyen et al. 2025a); *S. ouboteri* Pham, Pham, Le, Ngo, Ngo, Ziegler & Nguyen, 2024 from Phu Tho Province (Pham et al. 2024); and *S. truongi* Pham, Ziegler, Pham, Hoang, Ngo & Le, 2025 from Son La Province (Pham et al. 2025).

During our fieldwork in the evergreen forests of Dak Lak Province, Vietnam, a new population of forest skinks was found in Krong Trai Nature Reserve (NR). The skinks could be assigned to the genus *Scincella* based on diagnostic morphological characteristics (Smith 1935; Taylor 1963; Ouboter 1986; Nguyen et al. 2010b). In-depth morphological comparisons in concert with molecular analyses showed that they are distinct from all other existing species. Subsequent phylogenetic analyses based on the mitochondrial COI gene confirmed their placement within the genus *Scincella* and revealed that they form a strongly corroborated clade with *S. auranticaudata*, *S. badenensis*, *S. nigrofasciata*, *S. rufocaudata*, and *S. rupicola*. The statistical analyses (MFA) based on morphological comparisons of scalation, coloration, and morphometric characters further supported their distinction from *S. auranticaudata*, *S. badenensis*, *S. nigrofasciata*, *S. rufocaudata*, and *S. rupicola*, and all other congeners. We therefore describe this population as a new species of *Scincella* from Dak Lak Province.

## Materials and methods

### Sampling

A field survey was conducted in May 2023 in Krong Trai NR, Dak Lak Province, Vietnam by D.T. Do, T.Q. Phan, C.T. Pham, T.Q. Nguyen, C.V. Hoang, H.N. Ngo (hereafter Do et al.). Specimens were collected between 19:00 and 22:00. After having been photographed in life, skinks were anaesthetized and euthanized in a closed vessel with a piece of cotton wool containing ethyl acetate (Simmons 2002), fixed in 85% ethanol for ten hours, and then later transferred to 75% ethanol for permanent storage. Tissue samples were preserved separately in 70% ethanol before fixation. Voucher specimens were deposited in the collections of the Institute of Biology (**IB**), Hanoi, Vietnam.

### Molecular data and phylogenetic analyses

We sequenced two samples of the new population from Dak Lak Province. Additionally, we included data of 37 COI sequences from 19 *Scincella* species from GenBank (Nguyen et al. 2025a; Pham et al. 2025) for phylogenetic analyses (Suppl. material 1). Three species, *Cryptoblepharus egeriae*, *Plestiodon elegans*, and *P. liui* were used as outgroup taxa. Tissue samples were extracted using DNeasy blood and tissue kit, Qiagen (Hilden, Germany). Extracted DNA from the fresh tissue was amplified by DreamTaq PCR mastermix, Thermo Fisher Scientific (Vilnius, Lithuania). A fragment of the mitochondrial cytochrome c oxidase subunit I (COI) was sequenced using the primer pair LCO1490 (5’-GGTCAACAAATCATAAAGATATTGG-3’) and HCO2198 (5’- TAAACTTCAGGGTGA CCAAAAAATCA-3’) (Folmer et al. 1994). The PCR reaction volume was 21 μl, including 10 μl of mastermix, 5 μl of water, 2 μl of each primer at 10 pmol/μl, and 2 μl of DNA or higher depending on the quantity of DNA in the final extraction solution. PCR condition was 95 °C for 5 min to activate the Taq polymerase; with 40 cycles at 95 °C for 30 s, 50 °C for 45 s, 72 °C for 60 s; and the final extension at 72 °C for 6 min. PCR products were subjected to electrophoresis through a 1% agarose gel, 1st BASE (Selangor, Malaysia). Gels were stained for 10 min in 1X TBE buffer at 2 pg/ml of ethidium-bromide and visualized under UV light. Successful amplifications were purified to eliminate PCR components using GeneJET™ PCR Purification Kit, Thermo Fisher Scientific (Vilnius, Lithuania). Purified PCR products were sent to Macrogen Inc. (Seoul, South Korea) for sequencing. Sequences generated in this study were edited using Geneious v. 7.1.8 (Kearse et al. 2012).

After aligned by Clustal X v. 2 (Thompson et al. 1997), data were analyzed using maximum parsimony (MP), as implemented in PAUP*4.0a169 (Swofford 2001), maximum likelihood (ML) as implemented in IQ-TREE v. 1.6.7.1 (Minh et al. 2020), and Bayesian inference (BI), as implemented in MrBayes v. 3.2.7 (Ronquist et al. 2012). For MP analysis, heuristic analysis was conducted with 100 random taxon addition replicates using tree-bisection and reconnection (TBR) branch swapping algorithm, with no upper limit set for the maximum number of trees saved. Bootstrap support was calculated using 1000 pseudo-replicates and 100 random taxon addition replicates. All characters were equally weighted and unordered. For the maximum likelihood (ML) analysis, we used a single model and 10,000 ultrafast bootstrap replications. The optimal model for nucleotide evolution was determined using jModeltest v. 2.1.4 (Darriba et al. 2012).

For Bayesian analyses, we used the optimal model selected by jModeltest with parameters estimated by MrBayes 3.2.1. Two independent analyses with four Markov chains (1 cold and 3 heated) were run simultaneously for ten million generations with a random starting tree and sampled every 1000 generations. Log-likelihood scores of sample points were plotted against generation time to determine stationarity of Markov chains. Trees generated before log-likelihood scores reached stationarity were discarded from the final analyses using the burn-in function. The posterior probability values for all clades in the final majority rule consensus tree were provided. The optimal model for nucleotide evolution was set to GTR+I+G for ML and single-model Bayesian analyses as selected by jModeltest v. 2.1.4. The cutoff point for the burn-in function was set to 25% of generated trees. Nodal support was also evaluated using bootstrap replication (BP) as estimated in PAUP, ultrafast bootstrap (UFB) in IQ-TREE, and posterior probabilities (PP) in MrBayes v. 3.2.7. BP ≥ 70; PP and UFB ≥ 0.95 were regarded as strong support for a clade (Hillis and Bull 1993; Ronquist et al. 2012; Hoang et al. 2018). Uncorrected pairwise divergences were calculated in PAUP*4.0a169.

### Morphological examination

Measurements were taken with a digital caliper (Electronic Digital Caliper) to the nearest 0.1 mm. The following morphological characteristics were recorded following Smith (1935), Taylor (1963), Grismer and Quah (2015), Pham et al. (2024, 2025), Bragin et al. (2025b), Nguyen et al. (2025a):

**SVL** snout-vent length (from tip of snout to cloaca);

**TaL** tail length (from cloaca to tip of tail);

**AG** distance from posterior junction of forelimb and body wall to anterior junction of hindlimb and body wall (with the limbs held at right angles to the body);

**HL** head length (from tip of snout to posterior margin of parietal or interparietal, depending on the longest distance);

**HW** head width (at the widest portion of temporal region);

**HH** head height (at the deepest portion of temporal region);

**SNL** snout length (from anterior margin of eye to tip of snout);

**STL** distance from snout to anterior border of tympanum;

**SFlL** snout-forelimb length (from the tip of the snout to the anterior axilla of the forelimb);

**END** distance from anterior margin of eye to posterior border of nostril;

**EL** eye length (distance between anterior and posterior corners of eyelid);

**PDD** palpebral disc diameter (maximum horizontal diameter of the palpebral disc);

**TYD** maximum diameter of tympanum;

**FlL** forelimb length (from anterior axilla of forelimb to the tip of fourth finger);

**HlL** hindlimb length (anterior groin of hindlimb and to the tip of fourth toe);

#### Scalation

The meristic data were recorded following Nguyen et al. (2025a) and Bragin et al. (2025b): **SO**: supraoculars; **NU**: enlarged nuchal scales (located immediately behind the parietal and upper temporal scales); **LO**: loreals; **SL**: supralabials; **IF**: infralabials; **PT**: primary temporals; **ST**: secondary temporals; **SC**: superciliaries; **PR**: preoculars (scales in contact with posterior loreal, superciliaries, and upper presubocular); **PRS**: presuboculars (scales in contact with posterior loreal, supralabials, and lower preocular); **PO**: postoculars (scales in contact with posterior supraocular, last supraciliars, primary temporals, and upper postsuboculars); **PSO**: postsuboculars (scales in contact with primary temporals, lower postoculars, and supralabials); **MBSR**: midbody scale rows; **DBR**: dorsal scale rows between dorsolateral stripes; **PVSR**: paravertebral scale rows (scales in a line from posterior edge of parietals to dorsal point opposite posterior margin of the medial precloacals); **VSR**: ventral scale rows (scale rows on venter between postmental and enlarged precloacal scales); **FL4**: subdigital lamellae on fourth finger; **TL4**: subdigital lamellae on fourth toe; **F-H**: the degree of contact between adpressed forelimb and hindlimb (0 = separated, 1 = overlapped); **F-E**: extent of contact between adpressed forelimbs and the posterior margin of eye (0 = not reaching, 1 = beyond); **PrC**: enlarged precloacal scales; **PF**: prefrontals; **CH**: chinshields. Bilateral scale counts were given as left/right.

#### Coloration

The coloration was recorded following Nguyen et al. (2011, 2025a) and Neang et al. (2018): **BB**: body bands; **BSD**: black spots on dorsum; **VBD**: vertebral black blotches; **LLT**: pale lateral stripe (above dark stripe); **DDS**: dark dorsolateral stripe; **LDS**: pale dorsolateral stripe; **LDSP**: pale dorsolateral spots; **DLS**: dark lateral spots; **DSUL**: dark spots on upper part of leg; **WSE**: white stripe on margins of eyelids; **DSTR**: dorsal stripes.

Sex identification was performed by inspection of presence or absence of hemipenes.

Morphological comparisons were based on data from the following literature: Gray (1838), Boulenger (1887), Günther (1896), van Denburgh (1912), Stejneger (1925), Barbour (1927), Smith (1935), Taylor (1963), Zhao and Huang (1982), Darevsky and Nguyen (1983), Ouboter (1986), Wang and Zhao (1986), Inger et al. (1990), Darevsky and Orlov (1997), Chen et al. (2001), Darevsky et al. (2004), Bourret (2009), Nguyen et al. (2010a, 2010b, 2010c), Pham et al. (2015, 2024, 2025), Neang et al. (2018), Nguyen et al. (2020, 2025a), Koizumi et al. (2022), Jia et al. (2023, 2024), Okabe et al. (2024), and Bragin et al. (2025a, 2025b).

### Statistical analyses

For the statistical analyses, the newly discovered population from Dak Lak Province was compared to their closest relatives based on the phylogeny of *Scincella* including *S. auranticaudata*, *S. badenensis*, *S. nigrofasciata*, and *S. rupicola*. Raw morphological data used for the analyses were obtained from the specimens collected in Krong Trai NR and from 16 specimens representing the four other *Scincella* species, available from previous studies (Taylor 1963; Neang et al. 2018; Nguyen et al. 2019, 2020). These raw data are presented in Suppl. material 2.

All statistical analyses were conducted in R v. 4.5.2 (R Core Team 2025). To remove the effects of allometry in the morphometric characters, morphometric data, and categorical morphological data were also normalized to adjust raw data of morphometrics using the following equation: Xadj = log(X) – ß[log(SVL)-log(SVLmean)], where Xadj = adjusted value; X = measured value; ß = unstandardized regression coefficient for each population and SVLmean = overall average SVL of all populations (Thorpe 1975, 1983; Turan 1999; Lleonart et al. 2000; Chan and Grismer 2025). A multiple factor analysis (MFA; Grismer 2025) was conducted to assess morphospace between individuals from Dak Lak Province and their close relatives using the GroupStruct2 (Grismer 2025). A non-parametric permutation multivariate analysis of variance (PERMANOVA) from PairwiseAdonis package in GroupStruct2 (Grismer 2025) was used to determine if the centroid locations and group clusters of each species were statistically different from each other based on the MFA load scores of dimensions 1–5 (Oksanen et al. 2020). An Euclidean (dis) similarity matrix was calculated using 50,000 permutations (Grismer 2025). A pairwise post hoc test was also applied to estimate the differences between the studied species pairs. A p-value of < 0.05 was considered to indicate a significant difference between the studied taxa.

## Results

### Phylogenetic analyses

**Figure 1.**
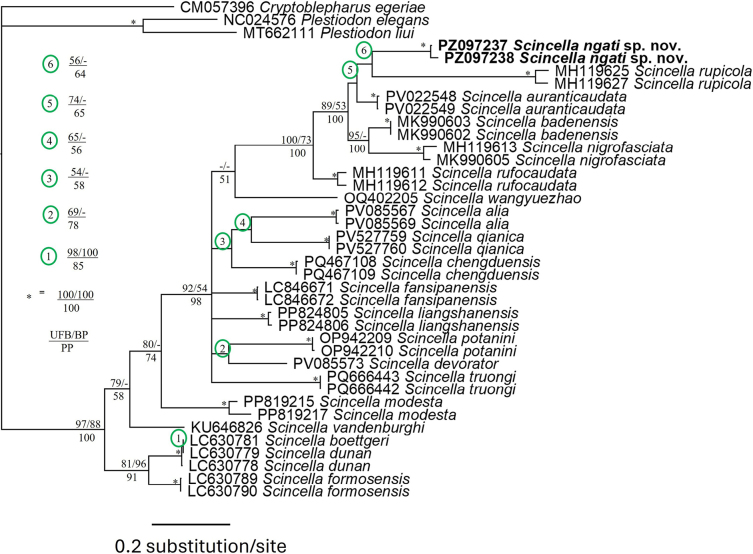
BI tree from a 629 bp sequence of the mitochondrial COI gene for *Scincella* and outgroup species; Bayesian posterior probabilities (BPP) and ML inferences bootstrap support value (UFB) are shown near the node. For GenBank accession numbers, refer to Suppl. material 1.

The final matrix of molecular data contained 629 characters with no gaps, of which 383 were parsimony informative. The MP analysis produced a single most parsimonious tree (Tree length = 1426, Consistency index = 0.31, Retention index = 0.65). Our phylogenetic results revealed that the new species forms a strongly supported clade (UFB = 89; BP = 53; PP = 1.0) with *S. auranticaudata*, *S. badenensis*, *S. nigrofasciata*, and *S. rupicola*, all of them occurring in south-central and southern Vietnam, with no obvious sister taxon (Fig. 1). In terms of genetic divergence, the new species is separated from *S. auranticaudata* and *S. rupicola* by 11.2–12.7% and 15.6–16.8%, respectively. It also differs from other species of *Scincella* incorporated in the study by at least 11.2% based on a fragment of the mitochondrial COI gene (Table 1).

**Table 1.** Uncorrected (“p”) distance matrix showing percentage pairwise genetic divergence (COI) between the new species (highlighted in bold) and closely related species.

			1	2	3	4	5	6	7	8	9	10	11	12	13	14	15	16	17	18	19	20	21	22	23	24	25	26	27	28	29	30	31	32	33	34	35	36	37	38	39
1	PZ097237	*Scincella ngati* sp. nov.	-																																						
2	PZ097238	*Scincella ngati* sp. nov.	1.34	-																																					
3	PV022548	*S. auranticaudata*	11.38	12.75	-																																				
4	PV022549	*S. auranticaudata*	11.20	12.57	0.48	-																																			
5	MK990603	*S. badenensis*	12.07	12.89	9.38	9.22	-																																		
6	MK990602	*S. badenensis*	12.07	12.89	9.38	9.22	0.00	-																																	
7	MH119613	*S. nigrofasciata*	11.95	12.72	12.40	11.92	10.81	10.81	-																																
8	MK990605	*S. nigrofasciata*	12.69	13.41	12.56	12.40	10.18	10.18	3.34	-																															
9	MH119611	*S. rufocaudata*	12.54	14.34	11.13	10.97	12.56	12.56	13.36	14.94	-																														
10	MH119612	*S. rufocaudata*	13.13	14.92	11.29	10.81	12.56	12.56	13.51	14.94	3.02	-																													
11	PV085567	*S. alia*	15.62	17.21	18.00	18.17	17.99	17.99	16.82	16.82	17.84	18.32	-																												
12	PV085569	*S. alia*	15.80	17.40	17.90	18.38	18.38	18.38	16.93	17.25	18.24	19.04	0.81	-																											
13	PQ467108	*S. chengduensis*	16.36	18.18	18.92	19.08	18.76	18.76	18.44	17.97	18.44	18.44	14.94	15.03	-																										
14	PQ467109	*S. chengduensis*	16.17	17.99	18.92	19.08	18.44	18.44	18.12	17.65	18.44	18.12	14.61	14.71	0.32	-																									
15	PV527759	*S. qianica*	17.85	18.21	18.44	18.92	18.12	18.12	20.19	19.24	17.97	17.17	16.07	16.00	16.38	16.06	-																								
16	PV527760	*S. qianica*	17.85	18.21	18.44	18.92	18.12	18.12	20.19	19.24	17.97	17.17	16.07	16.00	16.38	16.06	0.00	-																							
17	LC846671	*S. fansipanensis*	15.69	16.65	15.58	15.42	16.69	16.69	15.58	15.90	15.10	15.42	15.42	15.83	16.22	15.90	16.53	16.53	-																						
18	LC846672	*S. fansipanensis*	15.69	16.65	15.56	15.40	16.69	16.69	15.57	15.88	15.25	15.57	15.58	16.00	16.38	16.06	16.70	16.70	0.00	-																					
19	PP824805	*S. liangshanensis*	17.21	18.28	18.92	19.08	17.81	17.81	17.33	17.65	16.22	16.22	15.90	15.98	15.90	15.58	14.31	14.31	12.24	12.20	-																				
20	PP824806	*S. liangshanensis*	17.40	18.47	19.56	19.71	17.81	17.81	17.65	17.65	16.22	15.90	16.38	16.47	15.74	15.42	14.31	14.31	12.72	12.68	1.11	-																			
21	OP942209	*S. potanini*	16.38	17.50	18.76	18.92	18.44	18.44	16.85	17.49	17.33	17.17	17.19	17.92	16.85	16.53	17.01	17.01	14.94	14.94	13.83	14.15	-																		
22	OP942210	*S. potanini*	16.57	17.67	18.76	18.92	18.60	18.60	17.01	17.65	17.33	17.17	17.51	18.24	17.01	16.69	17.17	17.17	15.10	15.11	14.15	14.47	0.32	-																	
23	PV085573	*S. devorator*	15.53	17.37	16.22	16.38	17.81	17.81	18.25	18.08	16.37	16.38	17.36	17.16	15.56	15.24	15.57	15.57	14.11	14.09	15.56	15.41	14.60	14.92	-																
24	PQ666443	*S. truongi*	19.07	20.62	17.17	17.65	17.49	17.49	18.76	18.44	18.28	17.65	16.73	16.79	16.22	15.90	17.81	17.81	16.38	16.37	17.97	17.97	18.76	19.08	17.67	-															
25	PQ666442	*S. truongi*	19.07	20.62	17.17	17.65	17.49	17.49	18.76	18.44	18.28	17.65	16.73	16.79	16.22	15.90	17.81	17.81	16.38	16.37	17.97	17.97	18.76	19.08	17.67	0.00	-														
26	LC630781	*S. boettgeri*	19.87	21.02	20.19	20.35	19.40	19.40	19.87	19.40	19.24	18.12	18.95	19.35	18.28	17.97	20.19	20.19	18.92	18.95	19.71	19.40	18.76	18.92	17.82	18.76	18.76	-													
27	LC630779	*S. dunan*	19.87	21.02	20.19	20.35	19.40	19.40	19.87	19.40	19.24	18.12	18.95	19.35	18.28	17.97	20.19	20.19	18.92	18.95	19.71	19.40	18.76	18.92	17.82	18.76	18.76	0.00	-												
28	LC630778	*S. dunan*	19.69	20.84	20.03	20.19	19.24	19.24	19.71	19.24	19.08	17.97	18.79	19.19	18.12	17.81	20.03	20.03	18.76	18.79	19.56	19.24	18.60	18.76	17.66	18.92	18.92	0.16	0.16	-											
29	LC630789	*S. formosensis*	19.93	20.18	19.56	19.40	19.87	19.87	20.03	20.19	19.87	19.56	18.32	18.39	17.97	17.97	19.56	19.56	19.24	19.27	19.40	19.24	18.92	19.08	17.67	18.12	18.12	9.54	9.54	9.38	-										
30	LC630790	*S. formosensis*	19.92	20.17	19.56	19.40	19.87	19.87	20.03	20.19	19.87	19.56	18.31	18.39	17.97	17.97	19.56	19.56	19.24	19.27	19.40	19.24	18.92	19.08	17.66	18.12	18.12	9.38	9.38	9.22	0.16	-									
31	KU646826	*S. vandenburghi*	20.45	21.86	19.24	19.40	20.83	20.83	18.60	19.56	19.08	18.60	17.66	18.39	16.22	15.90	19.40	19.40	17.49	17.36	16.53	16.85	17.33	17.49	17.97	17.17	17.17	14.31	14.31	14.15	14.15	14.15	-								
32	PP819215	*S. modesta*	19.20	21.40	18.44	18.92	19.40	19.40	18.60	18.28	19.56	19.08	18.79	18.71	17.81	17.49	18.60	18.60	16.38	16.22	16.53	16.85	16.85	17.01	17.01	16.85	16.85	15.26	15.26	15.42	17.17	17.17	15.26	-							
33	PP819217	*S. modesta*	20.88	22.31	18.76	19.24	19.56	19.56	19.40	18.92	20.35	20.03	18.80	18.87	19.08	18.76	20.19	20.19	16.53	16.37	17.17	17.49	17.33	17.49	16.86	17.81	17.81	17.17	17.17	17.01	16.69	16.69	15.58	5.25	-						
34	OQ402205	*S. wangyuezhaoi*	16.97	18.40	17.97	18.12	17.81	17.81	17.33	17.33	16.69	16.69	18.15	18.87	17.65	17.33	16.06	16.06	16.22	15.91	16.53	16.53	16.53	16.85	17.17	19.40	19.40	19.71	19.71	19.56	19.71	19.71	16.85	15.10	17.81	-					
35	MH119625	*S. rupicola*	16.12	16.81	18.28	18.12	17.49	17.49	16.69	16.69	18.60	19.08	19.68	19.82	18.12	17.81	20.67	20.67	18.92	18.97	19.24	18.92	18.76	18.76	19.84	21.15	21.15	20.51	20.51	20.67	21.46	21.30	20.83	19.87	21.30	17.97	-				
36	MH119627	*S. rupicola*	15.61	16.34	17.81	17.65	17.65	17.65	17.01	16.69	19.08	19.56	19.72	19.98	18.76	18.44	20.19	20.19	19.24	19.28	20.03	19.71	18.28	18.44	19.08	22.42	22.42	21.15	21.15	21.30	22.10	21.94	21.46	20.19	21.46	18.76	4.13	-			
37	CM057396	*Cryptoblepharus egeriae*	20.36	21.17	20.83	21.30	21.46	21.46	21.46	21.30	21.30	20.67	22.00	22.53	24.01	23.69	22.89	22.89	20.67	20.55	21.30	21.30	22.58	22.89	21.81	21.78	21.78	18.92	18.92	18.76	20.03	20.03	20.83	20.83	21.30	21.46	21.78	22.10	-		
38	NC024576	*Plestiodon elegans*	19.97	20.07	20.67	20.51	21.94	21.94	21.46	20.67	21.78	21.15	22.49	22.39	22.58	22.58	22.73	22.73	21.46	21.35	22.58	22.73	22.26	22.58	22.98	23.53	23.53	21.46	21.46	21.62	20.83	20.83	20.99	20.35	21.46	21.94	21.94	21.30	21.78	-	
39	MT662111	*Plestiodon liui*	22.76	24.03	21.15	21.30	21.15	21.15	21.94	20.99	23.69	22.89	23.30	23.19	21.94	21.94	22.26	22.26	20.83	20.70	21.78	21.46	21.46	21.78	21.04	21.94	21.94	21.94	21.94	22.10	22.58	22.58	22.58	20.51	20.67	21.15	22.42	22.26	20.19	16.53	-

### Statistical analyses

**Figure 2.**
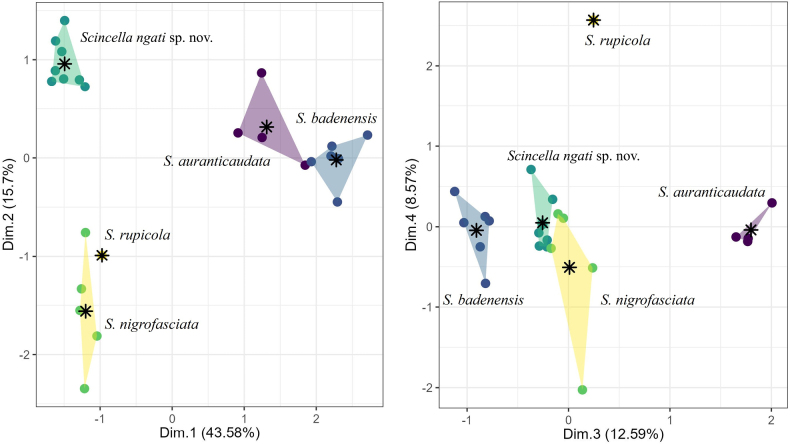
MFA scatter plots showing the morphospatial relationships among selected the *Scincella* species along the first four dimensions

In the MFA analysis, the four dimensions explain 43.58%, 15.7%, 12.59%, and 8.57% of the total variation, respectively, accounting for 80.44% of the total variation in the dataset (Fig. 2). The MFA analysis revealed that, although the Krong Trai NR population overlaps with *S. nigrofasciata*, *S. auranticaudata*, and *S. badenensis* in the fourth dimension, they are separated from each other in the first three dimensions, which account for 71.87% of the total variation (Fig. 2). Additionally, the Krong Trai NR population is also separated from *S. rupicola* along the ordination of the first four dimensions. The PERMANOVA analysis also indicated that the new population in Krong Trai NR differs significantly in morphospace from closely related species of *Scincella*, except for *S. rupicola* due to only a single individual from Thailand (Taylor 1963) was included in our analysis (Table 2).

**Table 2.** Summary statistics from the PERMANOVA analysis from the loadings of the MFA comparing *Scincella ngati* sp. nov. to closely related species.

Comparison	R2	F.value	p.value
*Scincella ngati* sp. nov. vs *S. auranticaudata*	0.7826	35.9982	0.002
*Scincella ngati* sp. nov. vs *S. badenensis*	0.7867	44.2663	< 0.001
*Scincella ngati* sp. nov. vs *S. nigrofasciata*	0.5129	11.5838	0.001
*Scincella ngati* sp. nov. vs *S. rupicola*	0.7187	17.8844	0.1123

### Taxonomic account

#### Scincella ngati sp. nov.

Taxon classificationAnimaliaSquamataScincidae

5DB78964-C3C0-529D-B06A-B66E078B5773

https://zoobank.org/046DAFE3-F30E-4EF9-9BDD-BC2D688E962A

Figs 3, 4, 5, 6

##### Material examined.

**Figure 3.**
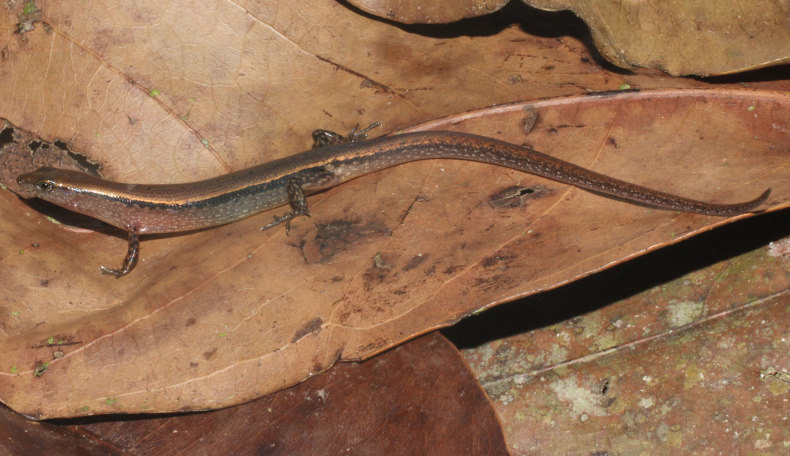
Holotype of *Scincella ngati* sp. nov. (IB R.6445) in life, adult male.

**Figure 4.**
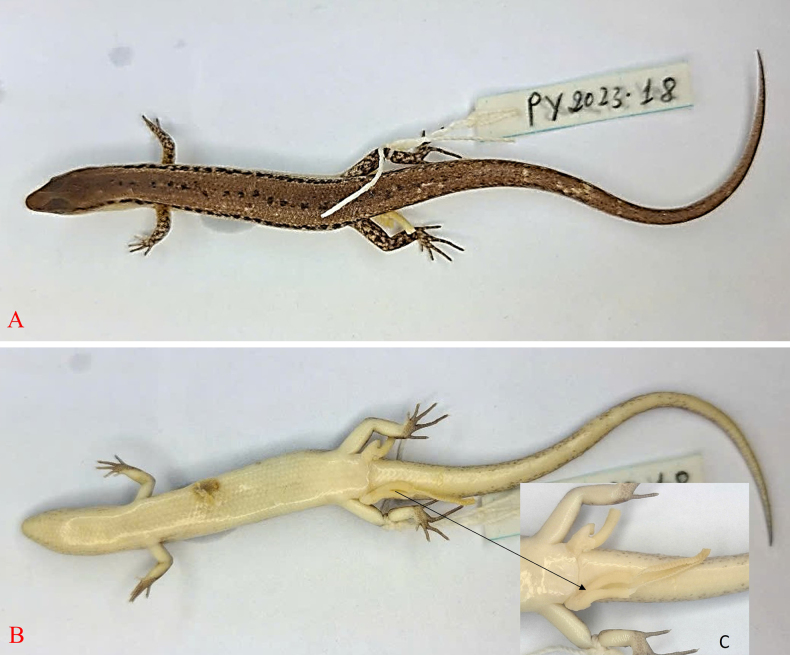
Holotype of *Scincella ngati* sp. nov. (IB R.6445) in preservative. **A**. Dorsal view; **B**. Ventral view; **C**. Hemipenes in preservative.

**Figure 5.**
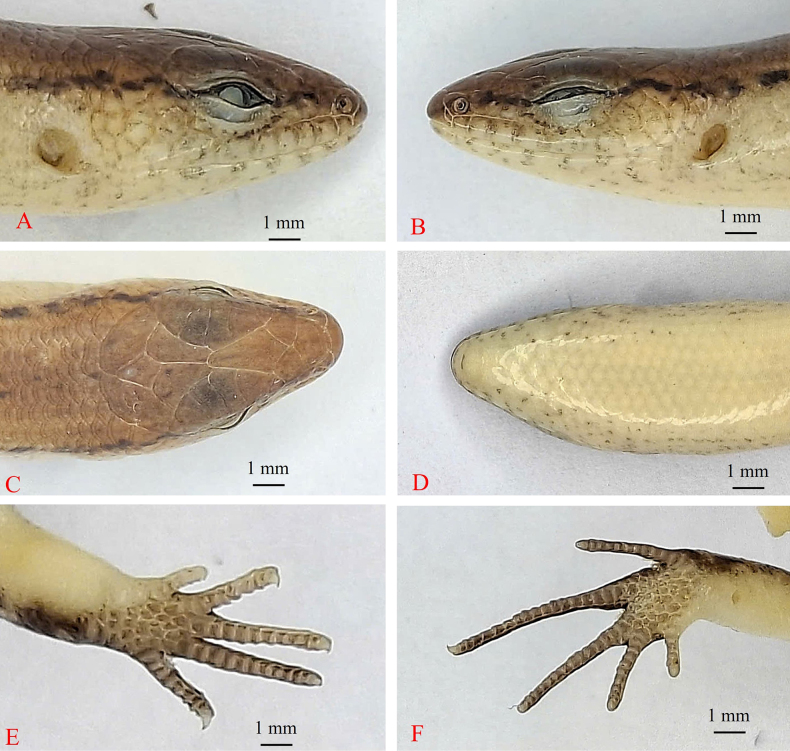
Holotype of *Scincella ngati* sp. nov. (IB R.6445). **A**. Lateral right view of head; **B**. Lateral left view of head; **C**. Dorsal view of head; **D**. Ventral view of head; **E**. Ventral view of forelimb; **F**. Ventral view of hindlimb.

***Holotype***. • IB R.6445 (Field number PY2023.18) (Figs 3, 4, 5), adult male, collected on 20 May 2023 by Do et al. in the evergreen forest of Krong Trai NR (13.84157, 108.57566, at an elevation of 232 m a.s.l.), Dak Lak Province, Vietnam. ***Paratypes***. • IB R.6446 (Field number PY2023.17), adult male; • IB R.6447 (Field number PY2023.19), adult male; • IB R.6448 (Field number PY2023.108), adult male; • IB R.6449 (Field number PY2023.16); • adult female, IB R.6450 (Field number PY2023.20); • adult female, IB R.6451 (Field number PY2023.21); • adult female, IB R.6452 (Field number PY2023.109); • adult female, all with the same data as the holotype.

**Figure 6.**
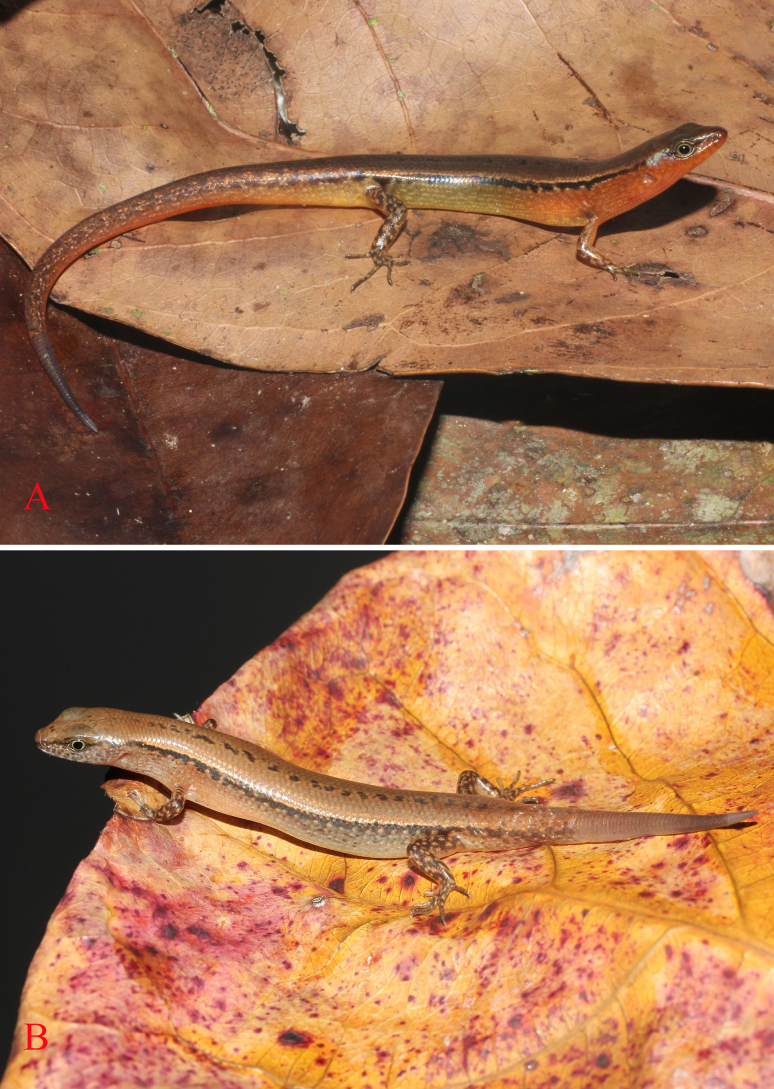
Paratypes of *Scincella ngati* sp. nov. in life. **A**. Dorsolateral view (IB R. 6447, male) **B**. Dorsolateral view (IB R. 6452, female).

##### Diagnosis.

The new species can be distinguished from other species of *Scincella* by a combination of the following characteristics: size medium (SVL ≤ 48.3 mm); primary temporals two; external ear opening without lobules; loreals two; supralabials seven (rarely eight); infralabials six; enlarged nuchals 0–2 on each side; midbody scales in 32–34 rows; dorsal scales smooth, in eight rows across the back; paravertebral scales 68–70, not widened; ventral scales in 64–68 rows; 10 or 11 smooth lamellae beneath finger IV and 16 or 17 beneath toe IV; toes not reaching the fingers when limbs adpressed along body; dorsal surface of body and tail bronze brown with a discontinuous black vertebral stripe, one scale wide, from middle of neck to tail base; a black stripe, two scales wide, interrupted by small pale spots, running from nostril to eye and extending from posterior margin of eye along upper part of flank and tail base.

##### Description of holotype.

Size medium (SVL 47.3 mm), tail long (TaL 69.5 mm); head longer than wide (HL 8.5 mm, HW 5.8 mm); snout obtuse, round anteriorly; rostral wider than high, distinctly visible from above; supranasals absent; frontonasal wider than long, in contact with rostral, nasals, anterior loreals, and frontal; prefrontals in contact with each other; frontal narrowing posteriorly, approximately 1.1 times longer than the distance to the tip of snout, in contact with prefrontals, first and second supraoculars, and frontoparietals; frontoparietals in contact with each other anteriorly, bordered by frontal, three supraoculars, parietals, and interparietal; interparietal lozenge-shaped, with a transparent spot posteriorly; parietals in contact posteriorly, posterolateral border surrounded by three scales on each side and one nuchal scale; enlarged nuchals 1/0; nostril in center of nasal, in contact with rostral, frontonasal, loreal, first supralabial; loreals two, anterior loreal higher but narrower than posterior one; preocular three; presuboculars three; supraciliaries 7/6; supraoculars four, the first longest, the second widest, fourth supraocular followed by a small posterior supraocular and a small postocular; postocular two; postsuboculars three; primary temporals two, lower one in contact with sixth and seventh supralabials; secondary temporals two, upper one large, in contact with posterolateral border of parietal, overlapped by lower one and parietal; lower eyelid with an undivided, bearing elongated, horizontally oriented transparent window opaque window (palpebral disc), separated from supralabials by two rows of granular scales; supralabials 8/7, fifth and sixth below the eye; external ear opening present, anterior margin with indistinct lobules, tympanum deeply sunk; mental wider than long, round anteriorly, in contact with anterior infralabials and postmental; infralabials six, first small; postmental undivided, in contact with mental, first infralabials on each side, and first pair of chinshields; chinshields in three pairs, first pair in contact with each other medially, second pair separated from each other by a gular scale, and third pair separated from each other by three scales; midbody scales in 34 rows; dorsal scales smooth, in eight rows across the back; paravertebral scales 70, not widened; ventral scales smooth, in 68 rows; precloacals four, inner scales overlapping outer ones, central two enlarged, left one overlapped by right one; tail thick at base, medial subcaudals slightly widened. Limbs relatively developed (FlL 0.25/SVL, HlL/SVL 0.38), pentadactyl, dorsal surface of digits covered by two scale rows on basal and by a single row on terminal phalanges; subdigital lamellae keeled, in one row under the digits, 11/11 under fourth finger and 17/17 under fourth toe; toes and fingers separated when adpressed along body, adpressed forelimb reaching the eye (Table 3).

**Table 3.** Morphological characteristics of *Scincella ngati* sp. nov. from Dak Lak Province, Vietnam.

Voucher	IB R.6445	IB R.6446	IB R.6447	IB R.6448	IB R.6449	IB R.6450	IB R.6451	IB R.6452	Min–Max
Type status	Holotype	Paratype	Paratype	Paratype	Paratype	Paratype	Paratype	Paratype	
Sex	M	M	M	M	F	F	F	F	
SVL	47.3	48.30	45	45.5	48.3	45.5	44.1	42.7	42.7–48.3
TaL	69.5	64.7	69.8	11.4*	60.8	65.4	60.9	30.2*	60.8–69.8
AG	25.3	24.9	24	24	25.5	23.7	23.5	22	22.0–25.5
SNL	4.0	4.3	3.9	3.8	4.1	4.2	3.6	3.5	3.5–4.2
STL	9.0	9.1	9.0	8.8	9.1	8.8	8.5	8.5	8.5–9.1
SFlL	17.6	17.6	16.7	17.2	17.8	17.2	16.5	16.5	16.5–17.8
ED	2.7	2.8	2.8	2.6	2.9	2.6	2.2	2.3	2.2–2.9
HL	8.5	8.6	8.3	8.5	8.6	8.2	8.1	8.0	8.0–8.6
HW	5.8	5.8	5.6	5.5	6.0	5.5	5.4	4.9	4.9–6
HH	4.9	4.7	4.2	4.6	4.5	4.8	4.2	3.9	3.9–4.9
TD	1.4	1.4	1.4	1.1	1.3	1.1	1.0	1.0	1–1.4
FlL	12	12.2	11.3	11.3	12.3	11.5	10.5	10.8	10.5–12.3
HLL	17.8	18.3	17	17.1	18.3	17.2	15.4	15.2	15.2–18.3
PDD	0.6	0.5	0.6	0.6	0.5	0.5	0.6	0.6	0.5–0.6
SO	4/4	4/4	4/4	4/4	4/4	4/4	4/4	4/4	4
NU	1/0	1/1	1/1	1/2	1/1	1/1	2/1	1/1	0–2
LO	2/2	2/2	2/2	2/2	2/2	2/2	2/2	2/2	2
PR	3/3	3/3	3/3	3/3	3/3	3/3	3/3	3/3	3
PRS	3/3	3/3	3/3	3/3	2/2	3/3	2/3	3/3	2–3
SC	7/6	7/7	7/7	7/7	7/6	7/7	7/7	7/7	7
PO	3/3	3/3	3/3	3/3	3/3	3/3	4/3	3/3	3–4
PSO	3/3	3/3	3/3	3/3	3/3	3/3	3/3	3/3	3
PT	2/2	1/2	2/2	2	2/2	2/2	2/2	2/2	1–2
ST	2/2	2/2	2/2	2/2	2/2	2/2	2/2	2/2	2
SL	8/7	7/7	7/7	7/7	7/7	7/7	7/7	7/7	7–8
IF	6/6	6/6	6/6	6/6	6/6	6/6	6/6	6/6	6
CH	3	3	3	3	3	3	3	3	3
MBSR	34	32	32	32	34	32	32	32	32–34
PVSR	70	67	69	68	70	70	70	70	68–70
DBR	8	8	8	8	8	8	8	8	8
VSR	68	64	68	64	67	69	66	64	64–68
PrC	2	2	2	2	2	2	2	2	2
FL4	11/11	10/10	10/11	10/10	10/10	10/10	10/10	10/10	10–11
TL4	17/17	16/16	16/16	17/17	17/17	17/17	17/16	16/16	16–17
PE	Yes	Yes	Yes	Yes	Yes	Yes	Yes	Yes	Yes
F-H	No	No	No	No	No	No	No	No	No
F-E	No	No	No	No	No	No	No	No	No
BB	No	No	No	No	No	No	No	No	No
BSD	No	No	No	No	No	No	No	No	No
VBD	Yes	Yes	Yes	Yes	Yes	Yes	Yes	Yes	Yes
LLT	Yes	Yes	Yes	Yes	Yes	Yes	Yes	Yes	Yes
DDS	Yes	Yes	Yes	Yes	Yes	Yes	Yes	Yes	Yes
LDS	Yes	Yes	Yes	Yes	Yes	Yes	Yes	Yes	Yes
LDSP	Yes	Yes	Yes	Yes	Yes	Yes	Yes	Yes	Yes
DLS	No	No	No	No	No	No	No	No	No
DSUL	Yes	Yes	Yes	Yes	Yes	Yes	Yes	Yes	Yes
WSE	Yes	Yes	Yes	Yes	Yes	Yes	Yes	Yes	Yes
DSTR	No	No	No	No	No	No	No	No	No

In preservative, the hemipenes are deeply forked with two symmetrical lobes, long. Lobes well-developed, round, with lateral orientation. When fully everted, the long lobes curve forward and outward and overlap at the ends; the rolled-out part has regular transverse grooves. Hemipenis bifurcating about 81% of its total length to base (Fig. 4C).

##### Coloration in life.

Dorsal surface of body and tail bronze brown with a discontinuous black vertebral stripe, from middle of neck to tail base; a black stripe, two scales wide, interrupted by small pale spots, running from nostril to eye and extending from posterior margin of eye along upper part of flank and tail base; a cream lateral stripe, edged above dark stripe, in one scale wide, running from nostril to eye and extending from posterior margin of eye along upper part of flank and tail base; supralabials and infralabials with dark bars on sutures; lateral side of the neck and flank pale grey with small bright spots; dorsal surface of limbs brown with pale spots; chin, throat, venter, underside of tail, underside of fore and hind limbs greyish cream (Fig. 3).

##### Coloration in preservative.

Dorsal surface of body and tail bronze brown with a discontinuous black vertebral stripe, one scale wide, from middle of neck to tail base; a black stripe, two scales wide, interrupted by small pale spots, running from nostril to eye and extending from posterior margin of eye along upper part of flank and tail base; supralabials and infralabials with dark bars on sutures; lateral side of the neck and flank pale grey; upper side of limbs brown with pale spots; chin, throat, venter, underside of tail, underside of fore and hind limbs greyish cream (Fig. 4).

##### Sexual dimorphism and variation.

The females differ from males in the absence of hemipenes. Most of the morphological characteristics of paratypes agree with those of the holotype; the following characters are variable: (1) midbody scales in 32 rows in seven paratypes (IB R.6447–6452); (2) paravertebral scale rows: 69 in IB R.6447, 68 in IB R.6448; (3) ventral scale rows: 64 in IB R.6448 and IB R.6452, 66 in IB R.6451, and 67 in IB R.6449; (4) enlarged nuchal: 1/1 in IB R.6446, IB R.6447, IB R.6447, IB R.6449, IB R.6450, and IB R.6452; 1/2 in IB R.6448 and IB R.6451; subdigital lamellae on fourth finger: 10/10 in IB R.6446, IB R.6448–52 and 10/11 in IB R.6447; subdigital lamellae on fourth toe: 16/16 in IB R.6446, IB R.6447, IB R.6452, and 17/16 in IB R.6451 (Table 3).

##### Distribution.

*Scincella ngati* sp. nov. is currently known only from the type locality in Krong Trai NR, Dak Lak Province, Vietnam.

##### Natural history.

**Figure 7.**
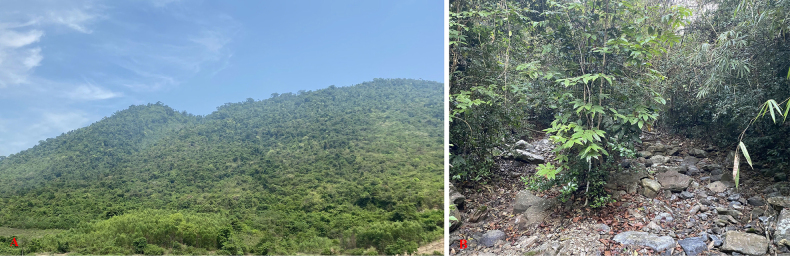
Habitat of *Scincella ngati* sp. nov. in Krong Trai Nature Reserve, Dak Lak Province, Vietnam. **A**. Evergreen forest; **B**. Microhabitat.

Specimens were found on the ground in leaf litter of evergreen forest between 19:00 and 22:00. The surrounding habitat was evergreen forest with medium and small hardwoods mixed with shrubs (Fig. 7). Air temperatures at the sites ranged from 26.8–33.5 °C and relative humidity was 60–78%. Other reptile species encountered at the sites included *Acanthosaura cuongi* Ngo, Le, Nguyen, Nguyen, Nguyen, Phan, Nguyen, Ziegler & Do; *Calotes bachae* Hartmann, Geissler, Poyarkov, Ihlow, Galoyan, Rödder, & Böhme; *C. versicolor* (Daudin), *Cyrtodactylus* sp.; *Dixonius vietnamensis* Das, and *Eutropis macularia* (Blyth).

##### Etymology.

We name the new species in honor of late Assoc. Prof. Dr. Ngat Nguyen Le from Hanoi National University of Education, in recognition of his contributions to the herpetofaunal exploration of Vietnam. We recommend “Ngat’s Smooth Skink” as the common English name and “Thằn lằn cổ ngật” as the common name in Vietnamese language for the new species.

##### Comparisons.

We compared the new species with other known taxa in the genus *Scincella* from Asia based on data obtained from the literature, and to their closest relatives based on the phylogeny of *Scincella*, including *S. auranticaudata*, *S. badenensis*, *S. nigrofasciata*, and *S. rupicola*. The new species is distinguished from *S. auranticaudata* by having a smaller size (males with maximal SVL 47.3 mm, *n* = 3 vs 62.1 mm, *n* = 2 and females with maximal SVL 48.3 mm, *n* = 5 vs 51.6 mm, *n* = 2), toes separated from fingers when limbs adpressed along body (vs overlapped), a smaller HL/SVL ratio (0.18–0.19 vs 0.21–0.25) and HW/SVL ratio (0.11–0.12 vs 0.14–0.15), and different dorsal color pattern (dorsum with one row of longitudinal black dots vs dorsum with two rows of longitudinal black dots); from *S. badenensis* by having a smaller size (males with maximal SVL 47.3 mm, *n* = 3 vs 64.4 mm, *n* = 4 and females with maximal SVL 48.3 mm, *n* = 5 vs 53.8 mm, *n* = 2), a greater ratio of HIL/SVL (0.37–0.39 vs 0.31–0.35), fewer lamellae beneath toe IV (16–17 vs 18–20), toes separated from fingers when limbs adpressed along body (vs overlapped), and different dorsal color pattern in males (dorsum with one row of longitudinal black dots vs absent); from *S. nigrofasciata* by having fewer ventral scales (64–68 vs 69–74), toes separated from fingers when limbs adpressed along body (vs overlapped), a greater % of bifurcated hemipenis (81% vs 63%), and different dorsal color pattern (dorsum with one row of longitudinal black dots vs dorsum with 5–7 regular discontinuous stripes); from *S. rupicola* by having a greater % of bifurcated hemipenis (81% vs 69–77%), toes separated from fingers when limbs adpressed along body (vs overlapped), and different dorsal color pattern (dorsum with one row of longitudinal black dots vs two rows of dark spots) (Tables 4, 5).

**Table 4.** Comparison of diagnostic morphometric (all in mm) characters of *Scincella ngati* sp. nov. to their closest relatives based on the phylogeny of *Scincella*. Abbreviations of morphological characters are provided in Materials and methods section. (n/a = not available).

	*Scincella ngati* sp. nov.	*S. auranticaudata*	*S. badenensis*	*S. nigrofasciata*	*S. rupicola*
SVL	42.7–48.3	48.9–62.1	47.8–64.4	40.0–52.6	34–55.2
TaL	60.8–69.8	61–85.3	74.4*	63.0–97.3	53.6–81.2
AG	22.0–25.5	24.4–31.4	23.4–33.8	20.1–29.7	19
HW	4.9–6	7.2–8.9	6.4–8.6	5.1–6.3	6
HD	3.9–4.9	n/a	n/a	3.8–4.5	n/a
ED	2.2–2.9	n/a	n/a	n/a	n/a
TD	1–1.4	1.2–1.8	1.4–1.7	1.3–1.6	n/a
SNL	3.5–4.2	18.4–23.2	n/a	3.0–3.8	n/a
SFIL	16.5–17.8	n/a	16.0–20.5	14.0–17.8	15.4
FLL	10.5–12.3	n/a	n/a	9.0–10.8	11
HLL	15.2–18.3	n/a	n/a	3.3–16.8	17
TaL/SVL	1.26–1.55	n/a	n/a	1.25–1.94	n/a
AGD/SVL	0.52–0.53	0.49–0.51	0.48–0.52	0.50–0.56	0.49
HW/SVL	0.11–0.12	0.14–0.15	0.13–0.15	n/a	0.16
HD/SVL	0.09–0.11	n/a	n/a	n/a	n/a
ED/SVL	0.05–0.06	n/a	n/a	n/a	n/a
TD/SVL	0.02–0.03	0.02–0.03	0.03	n/a	n/a
FLL/SVL	0.24–0.25	n/a	n/a	0.20–0.22	0.29
HLL/SVL	0.35–0.38	n/a	n/a	0.30–0.33	0.44
References	this study	Nguyen et al. (2025)	Nguyen et al. (2019)	Neang et al. (2018)	Smith (1916); Taylor (1963); Neang et al. (2018)

**Table 5.** Comparison of diagnostic meristic characters and color of *Scincella ngati* sp. nov. to their closest relatives based on the phylogeny of *Scincella*. Abbreviations of morphological characters are provided in Materials and Methods section. (n/a = not available).

	*Scincella ngati* sp. nov.	*S. auranticaudata*	*S. badenensis*	*S. nigrofasciata*	*S. rupicola*
PF	Yes	no (rare yes)		Yes	Yes
SO	4	4	4	4	4
NU	0.5–1.5	1	0–1	0–1	0–1
SC	7	8–9	8–9	7–8	7–9
PR	2	2	n/a	n/a	n/a
PRS	2–3	2	n/a	n/a	n/a
PO	3–4	n/a	n/a	n/a	n/a
PSO	3	n/a	n/a	n/a	n/a
PT	2 (rare 1)	2	2	2	n/a
ST	2	2	2–3	n/a	n/a
SL	7 (rare 8)	7	7–8	6–7	7
IF	6	6–7	6	6	6–7
MBSR	32–34	34–36	32–36	32–33	33–36
DBR	8	8		8	8
PVSR	68–70	67–74	67–71	69–74	68–73
VSR	64–68	65–69	68–74	65–69	63–69
PrC	2	2	2	2	n/a
FL4	10–11	10–13	8–11	10–11	n/a
TL4	16–17	17–20	18–20	15–17	17–21
F-H	No	Yes	Yes	Yes (rare no)	Yes
Coloration					
BB	No	No	No	No	Yes
BSD	No	Yes	Yes	Yes	Yes
VBD	Yes	Yes	No	Yes	Yes
LLT	Yes	Yes	No	Yes	Yes
DDS	Yes	Yes	No	Yes	Yes
LDS	Yes	No	No	Yes	Yes
LDSP	Yes	Yes	No	Yes	Yes
DLS	No	Yes	Yes	No	Yes
DSUL	Yes	Yes	Yes	Yes	Yes
WSE	Yes	No	No	No	Yes
DSTR	No	No	No	Yes	No
% of bifurcated hemipenis	81%	n/a	n/a	63%	69–77%
**References**	this study	Nguyen et al. (2025)	Nguyen et al. (2019)	Neang et al. (2018)	Smith (1916); Taylor (1963); Neang et al. (2018)

*Scincella ngati* sp. nov. has two primary temporals and thus differs from the following species in the genus *Scincella*: *S. alia*, *S. apraefrontalis*, *S. baraensis*, *S. balluca*, *S. darevskii*, *S. devorator*, *S. fansipanensis*, *S. honbaensis*, *S. melanosticta*, *S. monticola*, *S. punctatolineata*, and *S. rara*, which have only one primary temporal. The new species has toes separated from fingers when limbs adpressed along body, which differs from the following species, where toes and fingers overlapped: *S. baraensis*, *S. badenensis*, *S. dunan*, *S. formosensis*, *S. honbaensis*, *S. macrotis*, *S. melanosticta*, *S. ouboteri*, *S. reevesii*, and *S. rufocaudata*. In addition, the new species has the external ear opening without lobules and thus differs from the following taxa (with lobules): *S. boettgeri*, *S. darevskii*, *S. ochracea*, *S. ouboteri*, and *S. reevesii*.

The new species differs from *Scincella alia* by having more midbody scale rows (32–34 vs 26–28), more dorsal scale rows on back (8 vs 4), more paravertebral scales (68–70 vs 56–63), and fewer enlarged nuchals (0–2 vs 3); from *S. apraefrontalis* by having more midbody scale rows (32–34 vs 18), more paravertebral scales (68–70 vs 52), more ventral scales (64–68 vs 50), dorsal scales not enlarged (vs distinctly enlarged), more lamellae beneath toe IV (16–17 vs 8 or 9), and the presence of prefrontal (vs absent); from *S. balluca* by having smaller body size (maximal SVL 48.3 mm vs 57.9 mm), more paravertebral scales (68–70 vs 62–66), the presence of enlarged nuchals (vs absent), and fewer lamellae beneath toe IV (16–17 vs 18–20); from *S. baraensis* by having more midbody scale rows (32–34 vs 30), fewer enlarged nuchal (0–2 vs 3–4), and fewer lamellae beneath toe IV (16–17 vs 18–20); from *S. barbouri* by having fewer enlarged nuchals (0–2 vs 4–5), fewer ventral scales (64–68 vs 10–80), and more midbody scale rows (32–34 vs 26–28), from *S. boettgeri* by having smaller body size (maximal SVL 48.3 mm vs 56.9 mm) and more paravertebral scales (68–70 vs 59–66); from *S. capitanea* by having fewer enlarged nuchals (0–2 vs 3–4) and smaller body size (maximal SVL 48.3 mm vs 78.5 mm); from *S. chengduensis* by having larger body size (maximal SVL 48.3 mm vs 43.2 mm), more midbody scale rows (32–34 vs 23), more paravertebral scales (68–70 vs 57–60), and more lamellae beneath toe IV (16–17 vs 10–12); from *S. darevskii* by having smaller body size (maximal 48.3 mm vs 88.6 mm), more midbody scale rows (32–34 vs 28), fewer enlarged nuchals (0–2 vs 3), more paravertebral scales (68–70 vs 62), and fewer supraoculars (4 vs 5); from *S. devorator* by having more midbody scale rows (32–34 vs 28), fewer enlarged nuchals (0–2 vs 3), and more dorsal scale rows on back (8 vs 6); from *S. doriae* by having fewer enlarged nuchals (0–2 vs 4 or 5) and more dorsal scale rows on back (8 vs 6); from *S. dunan* by having more midbody scale rows (32–34 vs 26–29) and fewer enlarged nuchals (0–2 vs 3); from *S. fansipanensis* by having smaller body size (maximal 48.3 mm vs 59.0 mm) and more midbody scale rows (36 vs 22–24); from *S. huanrenensis* by having fewer ventral scales (64–68 vs 75–89) and more midbody scale rows (32–34 vs 26–28); from *S. liangshanensis* by having a smaller body size (maximal SVL 48.3 mm vs 61.9 mm), more lamellae beneath toe IV (16–17 vs 10–15), and more midbody scale rows (32–34 vs 23–27); from *S. macrotis* by having a larger body size (maximal SVL 48.3 mm vs 24.0 mm); from *S. melanosticta* by fewer supraciliaries (7 vs 8–9) and the presence of enlarged nuchals (vs absent); from *S. modesta* by having more midbody scale rows (32–34 vs 26–30) and fewer enlarged nuchals (0–2 vs 2–5); from *S. monticola* by having more midbody scale rows (32–34 vs 22–26), more dorsal scale rows on back (8 vs 4), and more ventral scales (64–68 vs 52–58); from *S. ochracea* by having more dorsal scale rows on back (8 vs 6); from *S. ouboteri* by having more dorsal scale rows on back (8 vs 6) and fewer enlarged nuchals (0–2 vs 2–4); from *S. qianica* by having more midbody scale rows (32–34 vs 26), more dorsal scale rows on back (8 vs 4), more lamellae beneath toe IV (16–17 vs 13–14), and enlarged fewer nuchals (0–2 vs 3); from *S. potanini* by having more midbody scale rows (32–34 vs 27); from *S. przewalskii* by having more supralabials (7 vs 6); from *S. punctatolineata* by having a larger body size (maximal SVL 48.3 mm vs 40.2 mm) and more midbody scale rows (32–34 vs 22–28); from *S. rara* by having more midbody scale rows (32–34 vs 24), more paravertebral scales (64–68 vs 53), and a single row of lamellae beneath toes II–IV (vs double rows); from *S. reevesii* by having toes separated fingers when limbs adpressed along body (vs overlapped); from *S. rufocaudata* by having toes separated from fingers when limbs adpressed along body (vs overlapped) and fewer dorsal scale rows on back (8 vs 10); from *S. schmidti* by having more midbody scale rows (32–34 vs 26) and more lamellae beneath toe IV (16–17 vs 11); from *S. truongi* by having more midbody scale rows (32–34 vs 28) and fewer enlarged nuchals (0–2 vs 3); from *S. tenuistriata* by having fewer enlarged nuchals (0–2 vs 2), a larger body size (maximal SVL 48.3 mm vs 42.4 mm), more midbody scale rows (32–34 vs 24), and more lamellae beneath toe IV (16–17 vs 11–12); from *S. tsinlingensis* by having fewer paravertebral scales (64–68 vs 70–90) and fewer ventral scales (64–68 vs 70–90); from *S. vandenburghi* by having more lamellae beneath toe IV (16–17 vs 12) and upper margin of lateral longitudinal stripe relatively straight (vs wavy); from *S. victoriana* by having more midbody scale rows (32–34 vs 26) and dorsal scales smooth (vs keeled); and from *S. wangyuezhaoi* by having fewer ventral scales (64–68 vs 71–89) and more midbody scale rows (32–34 vs 27–30).

## Discussion

The description of *Scincella ngati* brings the total number of species in the genus *Scincella* to 52. In addition, our discovery increases the number of *Scincella* species in Dak Lak Province to three, and in Vietnam to 22. The new species has a small known range with an estimate of less than 50 km^2^, which has been experiencing severe habitat degradation primarily due to road construction and timber logging. It is unclear whether these activities will significantly threaten its population, but they certainly affect the quality of its natural habitat. For the time being, we recommend listing the species as Data Deficient based on the IUCN Red List Categories and Criteria (IUCN 2025). Further research is needed to assess the population status of this species and to determine the impact of anthropogenic threats on the type locality and surroundings.

*Scincella rupicola* was described by Smith (1916) based on a single holotype specimen (BMNH 1946.8.16.77) from Siam (now Thailand). The species was subsequently recorded in Vietnam, Cambodia, and Laos (Teynié et al. 2004; Nguyen et al. 2010c; Neang et al. 2018). In Vietnam, this species is only known from Binh Thuan Province (now Lam Dong Province) (Bragin et al. 2025b). However, no reports on its morphological or molecular data from Vietnam has been published (Nguyen et al. 2009, 2010c). Morphological data of this species are not available for statistical analyses. In our phylogenetic tree, there are two sequences of *Scincella rupicola* collected from Mondulkiri Province, Cambodia (Neang et al. 2018). When compared with the holotype of *Scincella rupicola* from Siam, the new species can be distinguished from *S. rupicola* by having toes separated from fingers when limbs are adpressed along body (vs overlapped), a smaller HL/SVL ratio (0.18–0.19 vs 0.23), HW/SVL ratio (0.11–0.12 vs 0.16), FLL/SVL ratio (0.24–0.25 vs 0.29), HLL/SVL ratio (0.35–0.38 vs 0.44), and different dorsal color pattern (dorsum with one row of longitudinal black dots vs two rows of dark spots) (Taylor 1963).

## Supplementary Material

XML Treatment for Scincella ngati

## Additional information

### Use of AI

No use of AI was reported.

### Author ORCIDs

Anh Van Pham https://orcid.org/0000-0002-6023-3418

Dang Trong Do https://orcid.org/0000-0003-3048-1434

Truong Quang Nguyen https://orcid.org/0000-0002-6601-0880

Chung Van Hoang https://orcid.org/0000-0002-0709-974X

Mai Hong Thi Nguyen https://orcid.org/0009-0001-8322-010X

Minh Le https://orcid.org/0000-0002-2953-2815

Minh Duc Le https://orcid.org/0009-0000-5097-8531

Thomas Ziegler https://orcid.org/0000-0002-4797-609X

Cuong The Pham https://orcid.org/0000-0001-5158-4526

### Data availability

All of the data that support the findings of this study are available in the main text or Supplementary Information.

## Supplementary materials

Supplementary material 1 GenBank accession numbers Data type docx Explanation note GenBank accession numbers and associated samples used in this study. https://binary.pensoft.net/file/1574890This dataset is made available under the Open Database License (http://opendatacommons.org/licenses/odbl/1.0/). The Open Database License (ODbL) is a license agreement intended to allow users to freely share, modify, and use this Dataset while maintaining this same freedom for others, provided that the original source and author(s) are credited. Anh Van Pham, Dang Trong Do, Truong Quang Nguyen, Chung Van Hoang, Mai Hong Thi Nguyen, Minh Le, Minh Duc Le, Thomas Ziegler, Cuong The Pham

Supplementary material 2 Morphological characters Data type xlsx Explanation note Morphological characters of *Scincella ngati* sp. nov. and their closely related species. https://binary.pensoft.net/file/1574891This dataset is made available under the Open Database License (http://opendatacommons.org/licenses/odbl/1.0/). The Open Database License (ODbL) is a license agreement intended to allow users to freely share, modify, and use this Dataset while maintaining this same freedom for others, provided that the original source and author(s) are credited. Anh Van Pham, Dang Trong Do, Truong Quang Nguyen, Chung Van Hoang, Mai Hong Thi Nguyen, Minh Le, Minh Duc Le, Thomas Ziegler, Cuong The Pham
