# Supplementary material for: A new skink of the genus Scincella Mittleman, 1950 (Squamata, Scincidae) from Dak Lak Province, Vietnam
Source: Zookeys. 2026 Mar 31;1275:275–98. doi: 10.3897/zookeys.1275.178070 (PMC13058599; doi:10.3897/zookeys.1275.178070)
Supplement: Supplementary material 1 — GenBank accession numbers [file zookeys-1275-275_article-178070__-s001.docx]

**Supplementary material 1**.

**Table S1.** GenBank accession numbers and associated samples used in this study.

| **Voucher/Field number** | **Species** | **Locality** | **GenBank accession no.** | **References** |
| --- | --- | --- | --- | --- |
|  |  |  |  |  |
| IB R.6445 | *Scincella ngati* sp. nov. | Dak Lak, Vietnam | PZ097237 | This study |
| IB R.6446 | *Scincella ngati* sp. nov. | Dak Lak, Vietnam | PZ097238 | This study |
| ZMMU Re-18153 | *Scincella alia* | Tuyen Quang, Vietnam | PV085567 | Bragin et al. (2025b) |
| NAP-14094 | *Scincella alia* | Tuyen Quang, Vietnam | PV085569 | Bragin et al. (2025b) |
| ITBCZ 6527 | *Scincella auranticaudata* | Binh Thuan, Vietnam | PV022548 | Nguyen et al. (2025) |
| ITBCZ 7620 | *Scincella auranticaudata* | Binh Thuan, Vietnam | PV022549 | Nguyen et al. (2025) |
| ITBCZ 5966 | *Scincella badenensis* | Tay Ninh, Vietnam | MK990602 | Nguyen et al. (2019) |
| ITBCZ 5993 | *Scincella badenensis* | Tay Ninh, Vietnam | MK990603 | Nguyen et al. (2019) |
| KUZR68006 | *Scincella boettgeri* | Yonagunijima Island, Japan | LC630781 | Koizumi et al. (2022) |
| CIB 118786 | *Scincella chengduensis* | Sichuan, China | PQ467108 | Jia et al. (2025) |
| CIB 117637 | *Scincella chengduensis* | Sichuan, China | PQ467109 | Jia et al. (2025) |
| ZMMU NAP07169 | *Scincella devorator* | Ha Noi, Vietnam | PV085573 | Bragin et al. (2025b) |
| KUZ R65170 | *Scincella dunan* | Southern Ryukyus, Japan | LC630778 | Bragin et al. (2025b) |
| KUZR67027 | *Scincella dunan* | Southern Ryukyus, Japan | LC630779 | Bragin et al. (2025b) |
| IEBR R.5185 | *Scincella fansipanensis* | Lao Cai, Vietnam | LC846671 | Okabe et al. (2024) |
| IEBR R.5187 | *Scincella fansipanensis* | Lao Cai, Vietnam | LC846672 | Okabe et al. (2024) |
| KUZR37515 | *Scinella formosensis* | Taiwan | LC630789 | Koizumi et al. (2022) |
| KUZR37516 | *Scinella formosensis* | Taiwan | LC630790 | Koizumi et al. (2022) |
| XM-YXS80 | *Scincella liangshanensis* | Sichuan, China | PP824805 | Jia et al. (2024) |
| CIB 119513 | *Scincella liangshanensis* | Sichuan, China | PP824806 | Jia et al. (2024) |
| CIB 121415 | *Scincella modesta* | Zhejiang, China | PP819217 | Bragin *et al*. (2025b) |
| WYF11520 | *Scincella modesta* | Zhejiang, China | PP819215 | Bragin *et al*. (2025b) |
| ITBCZ 6344 | *Scincella nigrofasciata* | Ba Ria - Vung Tau, Vietnam | MK990605 | Nguyen et al. (2019) |
| CBC02545 | *Scincella nigrofasciata* | Mondulkiri, Cambodia | MH119613 | Neang et al. (2018) |
| DL-KD202109072 | *Scincella potanini* | Sichuan, China | OP942209 | Bragin et al. (2025b) |
| DL-KD202109071 | *Scincella potanini* | Sichuan, China | OP942210 | Bragin et al. (2025b) |
| QHU R2025001 | *Scincella qianica* | Guizhou, China | PV527759 | Xu et al. (2025) |
| QHU R2025002 | *Scincella qianica* | Guizhou, China | PV527760 | Xu et al. (2025) |
| ZMMU NAP-06163 | *Scincella rufocaudata* | Gia Lai, Vietnam | MH119611 | Neang et al. (2018) |
| ZMMU NAP-06164 | *Scincella rufocaudata* | Gia Lai, Vietnam | MH119612 | Neang et al. (2018) |
| S.r.-3 | *Scincella rupicola* | Preah Vihear, Cambodia | MH119625 | Neang et al. (2018) |
| S.r.-5 | *Scincella rupicola* | Krong Siem Reap, Cambodia | MH119627 | Neang et al. (2018) |
| HUS 2024.1 | *Scincella truongi* | Son La, Vietnam | PQ666442 | Pham *et al*. (2025) |
| IEBR R.6329 | *Scincella truongi* | Son La, Vietnam | PQ666443 | Pham *et al*. (2025) |
| G389SV | *Scincella vandenburghi* | Gangwon-do, South  Korea | KU646826 | Bragin et al. (2025b) |
| CIB 87246 | *Scincella wangyuezhao* | Sichuan, China | OQ402205 | Bragin et al. (2025b) |
| BTS541 | *Cryptoblepharus egeriae* | Christmas Is, Australia | CM057396 | Bragin et al. (2025b) |
| CB202004001 | *Plestiodon liui* | Jiangsu, China | MT.662111 | Bragin et al. (2025b) |
|  | *Plestiodon elegans* | China | NC024576 | Song et al. (2014) |

**Table S2.** Uncorrected (“p”) distance matrix showing percentage pairwise genetic divergence (COI) between the new species (highlighted in bold) and closely related species.

|  |  |  | 1 | 2 | 3 | 4 | 5 | 6 | 7 | 8 | 9 | 10 | 11 | 12 | 13 | 14 | 15 | 16 | 17 | 18 | 19 |
| --- | --- | --- | --- | --- | --- | --- | --- | --- | --- | --- | --- | --- | --- | --- | --- | --- | --- | --- | --- | --- | --- |
| 1 | PZ097237 | *Scincella ngati* sp. nov. | - |  |  |  |  |  |  |  |  |  |  |  |  |  |  |  |  |  |  |
| 2 | PZ097238 | *Scincella ngati* sp. nov. | 1.34 | - |  |  |  |  |  |  |  |  |  |  |  |  |  |  |  |  |  |
| 3 | PV022548 | *S. auranticaudata* | 11.38 | 12.75 | - |  |  |  |  |  |  |  |  |  |  |  |  |  |  |  |  |
| 4 | PV022549 | *S. auranticaudata* | 11.20 | 12.57 | 0.48 | - |  |  |  |  |  |  |  |  |  |  |  |  |  |  |  |
| 5 | MK990603 | *S. badenensis* | 12.07 | 12.89 | 9.38 | 9.22 | - |  |  |  |  |  |  |  |  |  |  |  |  |  |  |
| 6 | MK990602 | *S. badenensis* | 12.07 | 12.89 | 9.38 | 9.22 | 0.00 | - |  |  |  |  |  |  |  |  |  |  |  |  |  |
| 7 | MH119613 | *S. nigrofasciata* | 11.95 | 12.72 | 12.40 | 11.92 | 10.81 | 10.81 | - |  |  |  |  |  |  |  |  |  |  |  |  |
| 8 | MK990605 | *S. nigrofasciata* | 12.69 | 13.41 | 12.56 | 12.40 | 10.18 | 10.18 | 3.34 | - |  |  |  |  |  |  |  |  |  |  |  |
| 9 | MH119611 | *S. rufocaudata* | 12.54 | 14.34 | 11.13 | 10.97 | 12.56 | 12.56 | 13.36 | 14.94 | - |  |  |  |  |  |  |  |  |  |  |
| 10 | MH119612 | *S. rufocaudata* | 13.13 | 14.92 | 11.29 | 10.81 | 12.56 | 12.56 | 13.51 | 14.94 | 3.02 | - |  |  |  |  |  |  |  |  |  |
| 11 | PV085567 | *S. alia* | 15.62 | 17.21 | 18.00 | 18.17 | 17.99 | 17.99 | 16.82 | 16.82 | 17.84 | 18.32 | - |  |  |  |  |  |  |  |  |
| 12 | PV085569 | *S. alia* | 15.80 | 17.40 | 17.90 | 18.38 | 18.38 | 18.38 | 16.93 | 17.25 | 18.24 | 19.04 | 0.81 | - |  |  |  |  |  |  |  |
| 13 | PQ467108 | *S. chengduensis* | 16.36 | 18.18 | 18.92 | 19.08 | 18.76 | 18.76 | 18.44 | 17.97 | 18.44 | 18.44 | 14.94 | 15.03 | - |  |  |  |  |  |  |
| 14 | PQ467109 | *S. chengduensis* | 16.17 | 17.99 | 18.92 | 19.08 | 18.44 | 18.44 | 18.12 | 17.65 | 18.44 | 18.12 | 14.61 | 14.71 | 0.32 | - |  |  |  |  |  |
| 15 | PV527759 | *S. qianica* | 17.85 | 18.21 | 18.44 | 18.92 | 18.12 | 18.12 | 20.19 | 19.24 | 17.97 | 17.17 | 16.07 | 16.00 | 16.38 | 16.06 | - |  |  |  |  |
| 16 | PV527760 | *S. qianica* | 17.85 | 18.21 | 18.44 | 18.92 | 18.12 | 18.12 | 20.19 | 19.24 | 17.97 | 17.17 | 16.07 | 16.00 | 16.38 | 16.06 | 0.00 | - |  |  |  |
| 17 | LC846671 | *S. fansipanensis* | 15.69 | 16.65 | 15.58 | 15.42 | 16.69 | 16.69 | 15.58 | 15.90 | 15.10 | 15.42 | 15.42 | 15.83 | 16.22 | 15.90 | 16.53 | 16.53 | - |  |  |
| 18 | LC846672 | *S. fansipanensis* | 15.69 | 16.65 | 15.56 | 15.40 | 16.69 | 16.69 | 15.57 | 15.88 | 15.25 | 15.57 | 15.58 | 16.00 | 16.38 | 16.06 | 16.70 | 16.70 | 0.00 | - |  |
| 19 | PP824805 | *S. liangshanensis* | 17.21 | 18.28 | 18.92 | 19.08 | 17.81 | 17.81 | 17.33 | 17.65 | 16.22 | 16.22 | 15.90 | 15.98 | 15.90 | 15.58 | 14.31 | 14.31 | 12.24 | 12.20 | - |
| 20 | PP824806 | *S. liangshanensis* | 17.40 | 18.47 | 19.56 | 19.71 | 17.81 | 17.81 | 17.65 | 17.65 | 16.22 | 15.90 | 16.38 | 16.47 | 15.74 | 15.42 | 14.31 | 14.31 | 12.72 | 12.68 | 1.11 |
| 21 | OP942209 | *S. potanini* | 16.38 | 17.50 | 18.76 | 18.92 | 18.44 | 18.44 | 16.85 | 17.49 | 17.33 | 17.17 | 17.19 | 17.92 | 16.85 | 16.53 | 17.01 | 17.01 | 14.94 | 14.94 | 13.83 |
| 22 | OP942210 | *S. potanini* | 16.57 | 17.67 | 18.76 | 18.92 | 18.60 | 18.60 | 17.01 | 17.65 | 17.33 | 17.17 | 17.51 | 18.24 | 17.01 | 16.69 | 17.17 | 17.17 | 15.10 | 15.11 | 14.15 |
| 23 | PV085573 | *S. devorator* | 15.53 | 17.37 | 16.22 | 16.38 | 17.81 | 17.81 | 18.25 | 18.08 | 16.37 | 16.38 | 17.36 | 17.16 | 15.56 | 15.24 | 15.57 | 15.57 | 14.11 | 14.09 | 15.56 |
| 24 | PQ666443 | *S. truongi* | 19.07 | 20.62 | 17.17 | 17.65 | 17.49 | 17.49 | 18.76 | 18.44 | 18.28 | 17.65 | 16.73 | 16.79 | 16.22 | 15.90 | 17.81 | 17.81 | 16.38 | 16.37 | 17.97 |
| 25 | PQ666442 | *S. truongi* | 19.07 | 20.62 | 17.17 | 17.65 | 17.49 | 17.49 | 18.76 | 18.44 | 18.28 | 17.65 | 16.73 | 16.79 | 16.22 | 15.90 | 17.81 | 17.81 | 16.38 | 16.37 | 17.97 |
| 26 | LC630781 | *S. boettgeri* | 19.87 | 21.02 | 20.19 | 20.35 | 19.40 | 19.40 | 19.87 | 19.40 | 19.24 | 18.12 | 18.95 | 19.35 | 18.28 | 17.97 | 20.19 | 20.19 | 18.92 | 18.95 | 19.71 |
| 27 | LC630779 | *S. dunan* | 19.87 | 21.02 | 20.19 | 20.35 | 19.40 | 19.40 | 19.87 | 19.40 | 19.24 | 18.12 | 18.95 | 19.35 | 18.28 | 17.97 | 20.19 | 20.19 | 18.92 | 18.95 | 19.71 |
| 28 | LC630778 | *S. dunan* | 19.69 | 20.84 | 20.03 | 20.19 | 19.24 | 19.24 | 19.71 | 19.24 | 19.08 | 17.97 | 18.79 | 19.19 | 18.12 | 17.81 | 20.03 | 20.03 | 18.76 | 18.79 | 19.56 |
| 29 | LC630789 | *S. formosensis* | 19.93 | 20.18 | 19.56 | 19.40 | 19.87 | 19.87 | 20.03 | 20.19 | 19.87 | 19.56 | 18.32 | 18.39 | 17.97 | 17.97 | 19.56 | 19.56 | 19.24 | 19.27 | 19.40 |
| 30 | LC630790 | *S. formosensis* | 19.92 | 20.17 | 19.56 | 19.40 | 19.87 | 19.87 | 20.03 | 20.19 | 19.87 | 19.56 | 18.31 | 18.39 | 17.97 | 17.97 | 19.56 | 19.56 | 19.24 | 19.27 | 19.40 |
| 31 | KU646826 | *S. vandenburghi* | 20.45 | 21.86 | 19.24 | 19.40 | 20.83 | 20.83 | 18.60 | 19.56 | 19.08 | 18.60 | 17.66 | 18.39 | 16.22 | 15.90 | 19.40 | 19.40 | 17.49 | 17.36 | 16.53 |
| 32 | PP819215 | *S. modesta* | 19.20 | 21.40 | 18.44 | 18.92 | 19.40 | 19.40 | 18.60 | 18.28 | 19.56 | 19.08 | 18.79 | 18.71 | 17.81 | 17.49 | 18.60 | 18.60 | 16.38 | 16.22 | 16.53 |
| 33 | PP819217 | *S. modesta* | 20.88 | 22.31 | 18.76 | 19.24 | 19.56 | 19.56 | 19.40 | 18.92 | 20.35 | 20.03 | 18.80 | 18.87 | 19.08 | 18.76 | 20.19 | 20.19 | 16.53 | 16.37 | 17.17 |
| 34 | OQ402205 | *S. wangyuezhao* | 16.97 | 18.40 | 17.97 | 18.12 | 17.81 | 17.81 | 17.33 | 17.33 | 16.69 | 16.69 | 18.15 | 18.87 | 17.65 | 17.33 | 16.06 | 16.06 | 16.22 | 15.91 | 16.53 |
| 35 | MH119625 | *S. rupicola* | 16.12 | 16.81 | 18.28 | 18.12 | 17.49 | 17.49 | 16.69 | 16.69 | 18.60 | 19.08 | 19.68 | 19.82 | 18.12 | 17.81 | 20.67 | 20.67 | 18.92 | 18.97 | 19.24 |
| 36 | MH119627 | *S. rupicola* | 15.61 | 16.34 | 17.81 | 17.65 | 17.65 | 17.65 | 17.01 | 16.69 | 19.08 | 19.56 | 19.72 | 19.98 | 18.76 | 18.44 | 20.19 | 20.19 | 19.24 | 19.28 | 20.03 |
| 37 | CM057396 | *Cryptoblepharus egeriae* | 20.36 | 21.17 | 20.83 | 21.30 | 21.46 | 21.46 | 21.46 | 21.30 | 21.30 | 20.67 | 22.00 | 22.53 | 24.01 | 23.69 | 22.89 | 22.89 | 20.67 | 20.55 | 21.30 |
| 38 | NC024576 | *Plestiodon elegans* | 19.97 | 20.07 | 20.67 | 20.51 | 21.94 | 21.94 | 21.46 | 20.67 | 21.78 | 21.15 | 22.49 | 22.39 | 22.58 | 22.58 | 22.73 | 22.73 | 21.46 | 21.35 | 22.58 |
| 39 | MT662111 | *Plestiodon liui* | 22.76 | 24.03 | 21.15 | 21.30 | 21.15 | 21.15 | 21.94 | 20.99 | 23.69 | 22.89 | 23.30 | 23.19 | 21.94 | 21.94 | 22.26 | 22.26 | 20.83 | 20.70 | 21.78 |

Uncorrected ("p") distance matrix (continued)

|  |  |  | | 20 | 21 | 22 | 23 | 24 | 25 | 26 | 27 | 28 | 29 | 30 | 31 | 32 | 33 | 34 | 35 | 36 | 37 | 38 | 39 |
| --- | --- | --- | --- | --- | --- | --- | --- | --- | --- | --- | --- | --- | --- | --- | --- | --- | --- | --- | --- | --- | --- | --- | --- |
| 20 | PP824806 | | *S. liangshanensis* | - |  |  |  |  |  |  |  |  |  |  |  |  |  |  |  |  |  |  |  |
| 21 | OP942209 | *S. potanini* | | 14.15 | - |  |  |  |  |  |  |  |  |  |  |  |  |  |  |  |  |  |  |
| 22 | OP942210 | *S. potanini* | | 14.47 | 0.32 | - |  |  |  |  |  |  |  |  |  |  |  |  |  |  |  |  |  |
| 23 | PV085573 | *S. devorator* | | 15.41 | 14.60 | 14.92 | - |  |  |  |  |  |  |  |  |  |  |  |  |  |  |  |  |
| 24 | PQ666443 | *S. truongi* | | 17.97 | 18.76 | 19.08 | 17.67 | - |  |  |  |  |  |  |  |  |  |  |  |  |  |  |  |
| 25 | PQ666442 | *S. truongi* | | 17.97 | 18.76 | 19.08 | 17.67 | 0.00 | - |  |  |  |  |  |  |  |  |  |  |  |  |  |  |
| 26 | LC630781 | *S. boettgeri* | | 19.40 | 18.76 | 18.92 | 17.82 | 18.76 | 18.76 | - |  |  |  |  |  |  |  |  |  |  |  |  |  |
| 27 | LC630779 | *S. dunan* | | 19.40 | 18.76 | 18.92 | 17.82 | 18.76 | 18.76 | 0.00 | - |  |  |  |  |  |  |  |  |  |  |  |  |
| 28 | LC630778 | *S. dunan* | | 19.24 | 18.60 | 18.76 | 17.66 | 18.92 | 18.92 | 0.16 | 0.16 | - |  |  |  |  |  |  |  |  |  |  |  |
| 29 | LC630789 | *S. formosensis* | | 19.24 | 18.92 | 19.08 | 17.67 | 18.12 | 18.12 | 9.54 | 9.54 | 9.38 | - |  |  |  |  |  |  |  |  |  |  |
| 30 | LC630790 | *S. formosensis* | | 19.24 | 18.92 | 19.08 | 17.66 | 18.12 | 18.12 | 9.38 | 9.38 | 9.22 | 0.16 | - |  |  |  |  |  |  |  |  |  |
| 31 | KU646826 | *S. vandenburghi* | | 16.85 | 17.33 | 17.49 | 17.97 | 17.17 | 17.17 | 14.31 | 14.31 | 14.15 | 14.15 | 14.15 | - |  |  |  |  |  |  |  |  |
| 32 | PP819215 | *S. modesta* | | 16.85 | 16.85 | 17.01 | 17.01 | 16.85 | 16.85 | 15.26 | 15.26 | 15.42 | 17.17 | 17.17 | 15.26 | - |  |  |  |  |  |  |  |
| 33 | PP819217 | *S. modesta* | | 17.49 | 17.33 | 17.49 | 16.86 | 17.81 | 17.81 | 17.17 | 17.17 | 17.01 | 16.69 | 16.69 | 15.58 | 5.25 | - |  |  |  |  |  |  |
| 34 | OQ402205 | *S. wangyuezhao* | | 16.53 | 16.53 | 16.85 | 17.17 | 19.40 | 19.40 | 19.71 | 19.71 | 19.56 | 19.71 | 19.71 | 16.85 | 15.10 | 17.81 | - |  |  |  |  |  |
| 35 | MH119625 | *S. rupicola* | | 18.92 | 18.76 | 18.76 | 19.84 | 21.15 | 21.15 | 20.51 | 20.51 | 20.67 | 21.46 | 21.30 | 20.83 | 19.87 | 21.30 | 17.97 | - |  |  |  |  |
| 36 | MH119627 | *S. rupicola* | | 19.71 | 18.28 | 18.44 | 19.08 | 22.42 | 22.42 | 21.15 | 21.15 | 21.30 | 22.10 | 21.94 | 21.46 | 20.19 | 21.46 | 18.76 | 4.13 | - |  |  |  |
| 37 | CM057396 | *Cryptoblepharus egeriae* | | 21.30 | 22.58 | 22.89 | 21.81 | 21.78 | 21.78 | 18.92 | 18.92 | 18.76 | 20.03 | 20.03 | 20.83 | 20.83 | 21.30 | 21.46 | 21.78 | 22.10 | - |  |  |
| 38 | NC024576 | *Plestiodon elegans* | | 22.73 | 22.26 | 22.58 | 22.98 | 23.53 | 23.53 | 21.46 | 21.46 | 21.62 | 20.83 | 20.83 | 20.99 | 20.35 | 21.46 | 21.94 | 21.94 | 21.30 | 21.78 | - |  |
| 39 | MT662111 | *Plestiodon liui* | | 21.46 | 21.46 | 21.78 | 21.04 | 21.94 | 21.94 | 21.94 | 21.94 | 22.10 | 22.58 | 22.58 | 22.58 | 20.51 | 20.67 | 21.15 | 22.42 | 22.26 | 20.19 | 16.53 | - |

**Table S3.** Comparison of diagnostic morphometric (all in mm) characters of *Scincella ngati* sp. nov. to their closest relatives based on the phylogeny of *Scincella*. Abbreviations of morphological characters are provided in Materials and methods section. (n/a = not available).

|  | ***Scincella ngati* sp. nov.** | ***S. auranticaudata*** | ***S. badenensis*** | ***S. nigrofasciata*** | ***S. rupicola*** |
| --- | --- | --- | --- | --- | --- |
| SVL | 42.7–48.3 | 48.9–62.1 | 47.8–64.4 | 40.0–52.6 | 34–55.2 |
| TaL | 60.8–69.8 | 61–85.3 | 74.4* | 63.0–97.3 | 53.6–81.2 |
| AG | 22.0–25.5 | 24.4–31.4 | 23.4–33.8 | 20.1–29.7 | 19 |
| HW | 4.9–6 | 7.2–8.9 | 6.4–8.6 | 5.1–6.3 | 6 |
| HD | 3.9–4.9 | n/a | n/a | 3.8–4.5 | n/a |
| ED | 2.2–2.9 | n/a | n/a | n/a | n/a |
| TD | 1–1.4 | 1.2–1.8 | 1.4–1.7 | 1.3–1.6 | n/a |
| SNL | 3.5–4.2 | 18.4–23.2 | n/a | 3.0–3.8 | n/a |
| SFIL | 16.5–17.8 | n/a | 16.0–20.5 | 14.0–17.8 | 15.4 |
| FLL | 10.5–12.3 | n/a | n/a | 9.0–10.8 | 11 |
| HLL | 15.2–18.3 | n/a | n/a | 3.3–16.8 | 17 |
| TaL/SVL | 1.26–1.55 | n/a | n/a | 1.25–1.94 | n/a |
| AGD/SVL | 0.52–0.53 | 0.49–0.51 | 0.48–0.52 | 0.50–0.56 | 0.49 |
| HW/SVL | 0.11–0.12 | 0.14–0.15 | 0.13–0.15 | n/a | 0.16 |
| HD/SVL | 0.09–0.11 | n/a | n/a | n/a | n/a |
| ED/SVL | 0.05–0.06 | n/a | n/a | n/a | n/a |
| TD/SVL | 0.02–0.03 | 0.02–0.03 | 0.03 | n/a | n/a |
| FLL/SVL | 0.24–0.25 | n/a | n/a | 0.20–0.22 | 0.29 |
| HLL/SVL | 0.35–0.38 | n/a | n/a | 0.30–0.33 | 0.44 |
| References | this study | Nguyen et al. (2025) | Nguyen et al. (2019) | Neang et al. (2018) | Smith (1916); Taylor (1963); Neang et al. (2018) |

**Table S4.** Comparison of diagnostic metric characters and color of *Scincella ngati* sp. nov. to their closest relatives based on the phylogeny of *Scincella*. Abbreviations of morphological characters are provided in Materials and Methods section. (n/a = not available).

|  | ***Scincella ngati* sp. nov.** | ***S. auranticaudata*** | ***S. badenensis*** | ***S. nigrofasciata*** | ***S. rupicola*** |
| --- | --- | --- | --- | --- | --- |
| PF | Yes | no (rare yes) |  | Yes | Yes |
| SO | 4 | 4 | 4 | 4 | 4 |
| NU | 0.5–1.5 | 1 | 0–1 | 0–1 | 0–1 |
| SC | 7 | 8–9 | 8–9 | 7–8 | 7–9 |
| PR | 2 | 2 | n/a | n/a | n/a |
| PRS | 2–3 | 2 | n/a | n/a | n/a |
| PO | 3–4 | n/a | n/a | n/a | n/a |
| PSO | 3 | n/a | n/a | n/a | n/a |
| PT | 2 (rare 1) | 2 | 2 | 2 | n/a |
| ST | 2 | 2 | 2–3 | n/a | n/a |
| SL | 7 (rare 8) | 7 | 7–8 | 6–7 | 7 |
| IF | 6 | 6–7 | 6 | 6 | 6–7 |
| MBSR | 32–34 | 34–36 | 32–36 | 32–33 | 33–36 |
| DBR | 8 | 8 |  | 8 | 8 |
| PVSR | 68–70 | 67–74 | 67–71 | 69–74 | 68–73 |
| VSR | 64–68 | 65–69 | 68–74 | 65–69 | 63–69 |
| PrC | 2 | 2 | 2 | 2 | n/a |
| FL4 | 10–11 | 10–13 | 8–11 | 10–11 | n/a |
| TL4 | 16–17 | 17–20 | 18–20 | 15–17 | 17–21 |
| F-H | No | Yes | Yes | Yes (rare no) | Yes |
| Coloration |  |  |  |  |  |
| BB | No | No | No | No | Yes |
| BSD | No | Yes | Yes | Yes | Yes |
| VBD | Yes | Yes | No | Yes | Yes |
| LLT | Yes | Yes | No | Yes | Yes |
| DDS | Yes | Yes | No | Yes | Yes |
| LDS | Yes | No | No | Yes | Yes |
| LDSP | Yes | Yes | No | Yes | Yes |
| DLS | No | Yes | Yes | No | Yes |
| DSUL | Yes | Yes | Yes | Yes | Yes |
| WSE | Yes | No | No | No | Yes |
| DSTR | No | No | No | Yes | No |
| % of bifurcated hemipenis | 81% | n/a | n/a | 63% | 69-77% |
| **References** | this study | Nguyen et al. (2025) | Nguyen et al. (2019) | Neang et al. (2018) | Smith (1916); Taylor (1963); Neang et al. (2018) |
